# Cost-effectiveness of oral versus intravenous antibiotics (OVIVA) in patients with bone and joint infection: evidence from a non-inferiority trial

**DOI:** 10.12688/wellcomeopenres.15314.4

**Published:** 2020-01-08

**Authors:** Nicola McMeekin, Claudia Geue, Andrew Briggs, Ines Rombach, Ho Kwong Li, Philip Bejon, Martin McNally, Bridget L. Atkins, Jamie Ferguson, Matthew Scarborough, OVIVA collaborators

**Affiliations:** 1 HEHTA, Institute of Health and Wellbeing, University of Glasgow, Glasgow, G12 8RZ, UK; 2 Nuffield Department of Orthopaedics, Rheumatology and Musculoskeletal Sciences, University of Oxford, Oxford, OX3 7LD, UK; 3 Division of Infectious Diseases, Imperial College London, London, W12 0NN, UK; 4 Oxford University Hospitals NHS Foundation Trust, University of Oxford, Oxford, OX3 7HE, UK; 5 Nuffield Department of Medicine, University of Oxford, Oxford, OX3 7FZ, UK; 6 Wellcome Trust Research Programme, Kenya Medical Research Institute (KEMRI), Kilifi, Kenya; 7 The Bone Infection Unit, Nuffield Orthopaedic Centre, Oxford University Hospitals, Oxford, OX3 7HE, UK

**Keywords:** antibiotics, oral, intravenous, cost-effectiveness, non-inferiority, economic evaluation

## Abstract

**Background: **Bone and joint infections are becoming increasingly common and are usually treated with surgery and a course of intravenous antibiotics. However, there is no evidence to support the superiority of intravenous therapy and there is a growing body of literature showing that oral therapy is effective in treating these infections. Given this lack of evidence the clinical trial ‘Oral Versus Intravenous Antibiotics’ (OVIVA) was designed to assess the clinical and cost-effectiveness of intravenous versus oral antibiotics for the treatment of bone and joint infections, using a non-inferiority design. Clinical results from the trial indicate that oral antibiotics are non-inferior to intravenous antibiotics. The aim of this paper is to evaluate the cost-effectiveness of intravenous compared to oral antibiotics for treating bone and joint infections, using data from OVIVA.

**Methods: **A cost-utility analysis was carried out, the main economic outcome measure was the quality adjusted life-year, measured using the EQ-5D-3L questionnaire, combined with costs to estimate cost-effectiveness over 12-months follow-up.

**Results: **Results show that costs were significantly lower in the oral arm compared to the intravenous arm, a difference of £2,740 (95% confidence interval £1,488 to £3,992). Results of four sensitivity analyses were consistent with the base-case results. QALYs were marginally higher in the oral arm, however this difference was not statistically significant; -0.007 (95% confidence interval -0.045 to 0.031).

**Conclusions: **Treating patients with bone and joint infections for the first six weeks of therapy with oral antibiotics is both less costly and does not result in detectable differences in quality of life compared to treatment with intravenous antibiotics. Adopting a practice of treating bone and joint infections with oral antibiotics early in the course of therapy could potentially save the UK National Health Service over £17 million annually.

## Introduction

Bone and joint infections are becoming increasingly common. In the UK, the National Health Service (NHS) conducts around 190,000 hip and knee replacement surgeries annually; of these, approximately 1% will result in post-operative infections ^1, 2^. In addition, there are around 70,000 neck of femur fractures, surgery for which is associated with post-operative infection in up to 2.5% of cases, and 20,000 metalware or fracture-fixations with around a 15% infection rate (Personal communication, Dr M. Scarborough, opinion). There are also approximately 5,000 diabetic foot infections and a smaller number of infections of the axial skeleton annually. Treatment for these infections is estimated to cost around £20,000 to £40,000 per patient ^3– 5^.

These infections are usually treated with surgery and an initial course of intravenous antibiotics for 4–6 weeks. However, there is no evidence to support the superiority of intravenous therapy and, in recent years, there has been a growing body of literature showing that oral therapy is effective in treating these infections. A Cochrane review in 2013 ^6^ found there was no benefit of intravenous compared to oral antibiotics in treating bone and joint infection. The authors judged the trials to be of moderate to high risk of bias and there was no statistically significant difference in the pooled results. Furthermore, most of the trials were conducted over 20 years ago, when there was a lower prevalence of bone and joint infections. The authors concluded that there was insufficient evidence from this review to inform a change in practice and there was a need for a randomised controlled trial to investigate this further.

Intravenous treatment requires an access device to administer the antibiotic which carries risk of infection and thromboembolic disease. Oral antibiotics do not carry these risks, are less costly and more convenient. However, oral antibiotics have a higher risk of non-adherence and gastro-intestinal intolerance ^7^. Intravenous antibiotics are usually administered in a hospital setting but can be safely given in a clinic or at home, when administered outside the hospital this is called Outpatient Parenteral Antibiotic Therapy (OPAT). The OPAT team will visit the patient to administer the antibiotic, or the patient can choose to do this themselves. The OPAT team will oversee the patient’s care until the course of antibiotics is completed.

Given the lack of evidence on the superiority of intravenous compared to oral antibiotics, the clinical trial “OVIVA” was designed to assess the treatment failure rate and cost-effectiveness of intravenous versus oral antibiotics for the first six weeks treatment of bone and joint infections. The study directly tested the different antibiotic administration routes via a non-inferiority design set with a margin of 7.5 percentage points above the upper 90% confidence interval around the risk difference. Clinical results from the trial indicate that oral antibiotics are non-inferior to intravenous antibiotics. The primary clinical outcome of treatment failure (infection present) occurred in 74 of 506 participants (14.6%) in the intravenous arm and in 67 of 509 participants (13.2%) in the oral arm ^8^.

This paper reports on the within-trial cost-effectiveness of OVIVA, estimating cost and quality-adjusted life year (QALY) differentials comparing intravenous antibiotics to oral antibiotics for the first six weeks of treatment of bone and joint infections.

## Methods

### Overview of analysis

OVIVA was a UK based multi-centre, open-label, randomised, controlled non-inferiority trial with 12 months follow-up. Participants were adults (18+ years) who, in the attending clinician’s opinion, would normally be treated with a 6 weeks course of intravenous antibiotics for bone or joint infection. Participants started their randomised treatment within 7 days of surgery, or if no surgery for treatment of bone and joint infection, within 7 days of starting antibiotics. Participants were randomised to either intravenous or oral antibiotics for the first 6 weeks of therapy. In the intravenous arm, where it was common practice for adjunctive oral agents to be used alongside intravenous agents this was allowed. In the oral arm, if intravenous antibiotic treatment was needed for an unrelated illness, this was allowed for up to five days. Follow-on antibiotic treatment using either route of administration was allowed in both arms. Participants were recruited between June 2010 and October 2015. The primary endpoint was definite failure of infection treatment (infection present) within 12 months of randomisation. Treatment failure was identified locally by the treating clinician and categorised by a blinded end-point committee as: definite, probable and possible. The non-inferiority margin was set at 7.5%, and non-inferiority was met if the upper limit of the 90% CI around the absolute risk difference between the arms fell below this margin. Mortality was not necessarily considered a treatment failure in the absence of meeting criteria for a primary endpoint and was included in the secondary endpoint of ‘serious adverse events’. Full methodological details of the trial are available in the published protocol ^7^.

Individual patient data from the OVIVA trial were used to perform the cost-effectiveness analysis. Outcomes were measured in terms of QALYs. The analysis had a time horizon of 12 months and an NHS and personal social services perspective, reported in GBP sterling (2015 GBP). No discounting was needed due to the short time horizon. Best practice guidance was followed for conducting and reporting the analysis ^9, 10^. Cost-effectiveness was judged using incremental costs per health outcome measured against the current NICE threshold of £20,000 to £30,000. Missing resource and quality of life data were imputed using multiple imputation by chained equation ^11^ for the base case analysis and sensitivity analyses included a complete case analysis to explore the effect of excluding participants with missing data on the final results. Analysis was carried out in Stata 14.0 (StataCorp, College Station, TX, USA).

### Resource use

Resource use data were collected using self-reported questionnaires completed at 42, 120- and 365-days post randomisation. Resource use groups comprised: antibiotic medication, intravenous administration and inpatient stays. Antibiotic resource use included all antibiotics prescribed to each participant in the 12-month follow-up period. Inpatient stays were measured in bed days and intravenous administration included the cost of intravenous line insertion and removal for each intravenous episode per participant, cost of line complications where a new line is needed, and the cost of the Outpatient Parenteral Antimicrobial Therapy (OPAT) team if applicable.

Unit costs for antibiotic medication were obtained from the British National Formulary ^12^. Inpatient stays were valued using NHS reference costs ^13^ and intravenous administration resources and costs were taken from the literature ^14^ and expert opinion (Personal communication, Dr M. Scarborough). Costs were adjusted for inflation using the Hospital and Community Health Index ^15^. Unit costs and their sources are presented in Table 1.

**Table 1.** Unit costs and sources.

Resource	Unit cost	Source
Antibiotic	Various	British National Formulary ^12^
Inpatient stay	£296/overnight stay	NHS reference costs ^13^
*Intravenous administration*		
Insertion: PICC ^1^	£190	Expert opinion
Removal	£34	Expert opinion
*OPAT type*		
District nurse	£58 per hour	NHS reference costs ^13^
Inpatient (Hospital infusion centre)	£109 per hour	NHS reference costs ^13^

^1^Only 6 patients were reported to have a Hickman line inserted and the majority of patients had a PICC line. To be consistent within the IV arm, we assumed a constant cost for a PICC line for all patients. A Hickman line is likely to increase costs only marginally in the IV arm as these lines involve a surgeon’s time to be inserted. OPAT, outpatient parenteral antimicrobial therapy. Insertion is based on nursing time (Band 7/8a) and equipment used. Removal is based on 15 minutes nurse time plus equipment.

Total costs per participant were calculated by assigning unit costs to within trial resource use for each participant.

### Health outcomes

The economic outcome was the QALY, a measure combining both quality and length of life. Quality of life data were collected using the EQ-5D-3L questionnaire ^16^, administered at baseline, 14 days, 42 days, 120 days, 365 days. EQ-5D-3L responses were valued using a UK tariff ^17^. Standard area-under-the-curve methods were used to calculate QALYs ^18^, which were adjusted for baseline utility by including baseline utility as an explanatory variable ^19^.

### Missing data

Excluding participants with missing data can lead to loss of power and biased results because of a reduced sample size ^20^. Because of this, the missing data was analysed for type of missingness ^9, 11, 21^. Base-case data had missing resource and quality of life data; these missing data were imputed using multiple imputation by chained equation (MICE), which assumes data are missing at random ^11^. The effect of missing data was explored using both mean and multiple imputation. Missing cost values were imputed at the aggregate total cost level and missing quality of life data were replaced at utility score level at each EQ-5D-3L follow-up point using multiple imputation.

The regression analyses used to impute missing data included the same explanatory variables used in the missing data imputation in the clinical analysis ^8^.

### Assumptions

The following additional assumptions were made:

- As intervention resource use was not separately identified we have treated all resource use in the first 6-week period after randomisation as intervention resource use.
- The cost of a line insertion and removal was applied to the initial 6-week period of the intervention. In addition, it was assumed that an intravenous episode with a gap of two days or less between intravenous drugs did not require a new line to be inserted and a cost was not applied for insertion/removal. If the gap between episodes was greater than two days, it was assumed that a new line had to be inserted and the old line was removed, and a cost was assigned accordingly.
- The OPAT type recorded at the 42-day follow-up visit was used for each participant for all intravenous episodes in the 12-month follow-up period.
- Durations of antibiotics, intravenous episodes and inpatient stays per participant were truncated at 365 days.
- OPAT costs were applied at one hour per day when applicable.
- Where participants had an OPAT type of ‘inpatient’ and their intravenous episode extended beyond the inpatient stay, a weighted average cost of 2/5 Self-Administrating and 3/5 District Nurse was applied to the length of intravenous episode following discharge from hospital, this was the proportion of District Nurse to self-administering OPAT witnessed in the trial. The same weighted average was applied to participants with missing OPAT type.

### Data analysis

The base-case analysis used an intention to treat approach conducted on the multiple imputed dataset. Total mean costs, QALYs and associated standard errors were presented as well as the difference in total mean costs and QALYs between arms and a 95% confidence interval. Cost and QALY differences were estimated using a multivariate Generalised Linear Model with an identify link and Gauss distribution for QALY estimates and a Gamma distribution for cost estimation. In addition, covariates adjusted for in the QALY estimation were baseline utilities and age. An incremental cost-effectiveness ratio (ICER) is also presented; representing the difference in costs divided by the difference in QALYs. Participants with censored data (not due to death during the follow-up period) had costs and QALYs extrapolated using multiple imputation.

To explore the uncertainty around the cost and QALY differences and the resulting ICER, a non-parametric bootstrapping technique was employed with 1,000 iterations based on the unadjusted, non-imputed data. Results are presented using a cost-effectiveness plane, showing all 1,000 cost-effectiveness pairs.

The analysis was conducted using Stata version 14 (StataCorp. 2015. Stata Statistical Software: Release 14. College Station, TX: StataCorp LP.)

### Sensitivity analysis

Four sensitivity analyses were conducted: complete case analysis, mean imputation and two different assumptions for OPAT costs. Instead of using the above weighted average for participants with missing OPAT type, two scenarios were explored by varying the OPAT cost: applying solely the cost of a District Nurse, and applying solely the cost of Self-Administration. The sensitivity analyses results were analysed using the 2 sample t-test.

### Ethical approval

Research Ethics Committee Ref: 13/SC/0016 South Central Oxford REC B. Written informed consent was obtained from each participant by good clinical practice-trained research staff after assessing their understanding of the patient information sheet.

## Results

Baseline characteristics are presented in Table 2. The participants were well matched with no significant differences.

**Table 2.** Baseline characteristics of participants.

Characteristic	Intravenous (n=527)	Oral (n=527)	Total (n=1054)
**Age, years**			
Median (interquartile range)	61 (49–70)	60 (49–70)	60 (49–70)
Range	18–92	18–91	18–92
**Sex**			
Male, number (%)	320 (60.7)	358 (67.9)	678 (64.3)
Baseline surgical procedure, number (%)			
No implant or device present; debridement of chronic osteomyelitis performed	153 (29.0)	169 (32.1)	322 (30.6)
No implant or device present; debridement of chronic osteomyelitis not performed	25 (4.7)	29 (5.5)	54 (5.1)
Debridement and implant retention	124 (23.5)	123 (23.3)	247 (23.4)
Removal of orthopaedic device for infection	89 (16.9)	78 (14.8)	167 (15.8)
Prosthetic joint implant removed	68 (12.9)	67 (12.7)	135 (12.8)
Prosthetic joint implant, one-stage revision	47 (8.9)	43 (8.2)	90 (8.5)
Surgery for discitis, spinal osteomyelitis, or epidural abscess; debridement performed	8 (1.5)	5 (0.9)	13 (1.2)
Surgery for discitis, spinal osteomyelitis, or epidural abscess; debridement not performed	13 (2.5)	13 (2.5)	26 (2.5)

A total of 1,054 participants were recruited between June 2010 and October 2015; 527 in each arm, with 39 having no end-point data. In total, 23 participants died during the trial. Clinical results from the trial indicate that oral antibiotics are non-inferior to intravenous antibiotics with regards to definitive treatment failure. Treatment failure occurred in 74 of 506 participants (14.6%) in the intravenous arm and in 67 of 509 participants (13.2%) in the oral arm. The difference in risk, oral (PO) compared to intravenous (IV), of definitive failure in the intention-to-treat analysis was -1.4 percentage points (95% confidence interval, -5.6 to 2.9). These results were mirrored in the complete case intention-to-treat population, the per-protocol analysis (at least 4 weeks of randomised treatment received) and worst-case scenario analysis. These results are presented in more detail in the clinical trial paper ^8^.

### Resource use

Only 26 participants (2.5%) had missing resource use data; 12 in the intravenous arm and 14 in the oral arm. The results for complete case resource use are presented in Table 3, split between resources used in the initial 42-day intervention period and the remaining post-intervention period.

**Table 3.** Mean resource use per participant (complete case).

Resource type	Intravenous N=515 (97.7%)	Oral N=513 (97.3%)		
	Mean (SD)	Mean (SD)	Difference ‡	95% confidence interval
**Intervention period (Day 1 – 42)**				
Number of antibiotic prescriptions	3.53 (2.15)	3.53 (2.31)	0.002	-0.271 to 0.275
Antibiotic duration (days) * †	38.18 (32.64)	30.47 (29.12)	7.71	3.92 to 11.49
Number of inpatient admissions	1 (0)	1 (0)	0	N/A
Inpatient duration (days)	17.71 (14.83)	17.22 (18.62)	0.492	-1.57 to 2.55
Total number of days IV therapy was received * †	40.08 (30.52)	11.86 (27.45)	28.22	24.66 to 31.77
**Post-intervention period (Day 43 – 365)**				
Number of antibiotic prescriptions	3.17 (3.08)	2.90 (3.00)	0.274	-0.098 to 0.647
Antibiotic duration (days)	151.6 (181.6)	155.1 (161.2)	-3.53	-24.54 to 17.48
Number of inpatient admissions	0.829 (1.15)	0.821 (1.11)	0.008	-0.130 to 0.147
Inpatient duration (days)	8.51 (16.78)	9.13 (18.11)	-0.618	-2.76 to 1.52
Total number of days IV therapy was received	12.50 (34.83)	6.10 (18.49)	6.40	2.99 to 9.81
**Total**				
Number of antibiotic prescriptions	6.70 (3.74)	6.43 (3.93)	0.276	-0.194 to 0.746
Antibiotic duration (days)	189.8 (177.5)	185.6 (156.3)	4.18	-16.29 to 24.65
Number of inpatient admissions	1.83 (1.15)	1.82 (1.11)	0.01	-0.129 to 0.147
Inpatient duration (days)	26.22 (24.28)	26.35 (28.47)	-0.125	-3.36 to 3.11
Total number of days IV therapy was received	52.58 (40.37)	17.96 (33.52)	34.62	30.08 to 39.16

*The antibiotic duration sums the duration of all antibiotic use, including simultaneous use. For example, if a patient was on two different antibiotics for a period of five days, this would add to a duration of ten days. Intravenous duration includes the length of intravenous episodes where an intravenous line was needed to administer intravenous antibiotics (including more than one intravenous antibiotic taken at the same time as another).

^†^Antibiotic and IV therapy in the intervention period was not readily available from the data, the duration of these therapies were calculated by including all therapies finishing on or within 42 days of the start of first therapy treatment. SD, standard deviation; IV, intravenous ‡Difference between arms was calculated using t-tests

From the results in Table 3, it can be seen that for intervention resource use there was a statistically significant difference between arms in mean antibiotic and intravenous therapy duration. There were no statistically significant differences between arms in mean number of antibiotic prescriptions, number of inpatient admissions or inpatient duration. For resource use during the post-intervention period there was only a statistically significant difference between arms for intravenous therapy duration. This is mirrored at a total level where the only statistically significant difference between arms was for intravenous therapy; the mean total number of days for which intravenous therapy was received was 34.62 days longer in the intravenous arm. Table 4 presents the mean costs in both arms for unadjusted complete cases.

**Table 4.** Unadjusted costs (complete case).

Cost category	Intravenous N=515 (97.7%)	Oral N=513 (97.3%)		
	Mean (SD)	Mean (SD)	Difference ‡	95% confidence interval
**Intervention period (Day 1 – 42)**				
Antibiotics	£786 (£915)	£435 (£569)	£351	£257 to £443
Inpatient stays	£5,239 (£4,388)	£5,093 (£5,508)	£146	-£464 to £755
Intravenous costs	£2,950 (£2,555)	£1,231 (£1,304	£1,719	£1,471 to £1,968
**Total intervention costs**	**£8,974** **(£6,114)**	**£6,759** **(£6,196)**	**£2,215**	**£1,462 to £2,969**
**Post-intervention period (Day 43 – 365)**				
Antibiotics	£1,206 (£2,497)	£772 (£1,865)	£434	£164 to £704
Inpatient stays	£2,517 (£4,963)	£2,700 (£5,358)	-£183	-£815 to £449
Intravenous costs	£577 (£1,566)	£318 (£801)	£259	£107 to £412
**Total non-intervention**	**£4,301** **(£7,060)**	**£3,790** **(£6,899)**	**£511**	**-£343 to £1,366**
**Total**				
Antibiotics	£1,992 (£2,545)	£1,207 (£2,043)	£785	£502 to £1,067
Inpatient stays	£7,756 (£7,183)	£7,793 (£8,420)	-£37	-£995 to £920
Intravenous costs	£3,527 (£2,920)	£1,548 (£1,618)	£1,979	£1,690 to £2,268
**Total costs**	**£13,275** **(£10,113)**	**£10,549** **(10,371)**	**£2,727**	**£1,473 to £3,980**

SD, standard deviation. ‡Difference between arms was calculated using t-tests

The difference between arms in mean antibiotic and intravenous costs was statistically significant for intervention, post-intervention and total costs. However, there was only a statistically significant difference in mean total intervention costs, not for total post-intervention costs, £2,215 (95% CI £1,462 to £2,969) and £511 (95% CI -£343 to £1,366), respectively. This smaller and non-significant difference in total post-intervention costs is mainly due to lower intravenous costs after the initial 6-week intervention period.

The total mean cost combining intervention and non-intervention costs was £13,275 in the intravenous arm compared to £10,549 in the oral arm, a difference of £2,727, a statistically significant result.

Multiple imputation results reflect the complete case results presented above (a difference of £2,727), with intravenous mean costs £2,740 (£1,488 to £3,992) higher than in the oral arm, statistically significant.

### Health outcomes: QALYs

The utility values and missing data proportions for each follow-up point for the EQ-5D-3L questionnaire are presented in Table 5, along with complete case QALYs. For EQ-5D-5L results the proportion of missing data is similar in both arms. Participants in the oral antibiotic arm started from a slightly higher utility at baseline; 0.330 (SD 0.379) compared to 0.298 (SD 0.363). At the 14-day follow-up the mean utility was higher in the intravenous arm compared to the oral antibiotics arm; 0.437 (SD 0.304) compared to 0.421 (SD 0.338). The mean utilities for the remainder of the follow-up points revert to being higher in the oral arm. There were no statistically significant differences in mean utilities at any follow-up point. The utilities in both arms improved at each follow-up point compared to the previous one.

**Table 5.** EQ-5D-3L and quality adjusted life-years; complete cases.

Timepoint	EQ-5D-5L complete cases						Quality adjusted life-years complete cases					
	Intravenous		Oral				Intravenous		Oral			
	Mean (SD)	N (%)	Mean (SD)	N (%)	Difference (SE) ‡	95% confidence interval	Mean (SD)	N (%)	Mean (SD)	N (%)	Difference (SE) ‡	95% confidence interval
Baseline	0.298 (0.363)	386 (73.2%)	0.330 (0.379)	388 (73.6%)	-0.032 (0.027)	-0.085 to 0.020						
14 days	0.437 (0.304)	308 (58.4%)	0.421 (0.338)	309 (58.6%)	0.016 (0.026)	-0.035 to 0.067	0.014 (0.011)	297 (56.3%)	0.014 (0.012)	300 (56.9%)	-0.0001 (0.0009)	-0.0020 to 0.0017)
42 days	0.513 (0.316)	366 (69.4%)	0.531 (0.330)	374 (71.0%)	-0.018 (0.024)	-0.064 to 0.029	0.037 (0.022)	265 (50.3%)	0.037 (0.023)	274 (52.0%)	-0.0002 (0.0019)	-0.0039 to 0.0036
120 days	0.534 (0.337)	312 (59.2%)	0.544 (0.354)	306 (58.1%)	-0.011 (0.028)	-0.065 to 0.044	0.111 (0.063)	280 (53.1%)	0.116 (0.064)	279 (52.9%)	-0.0045 (0.0054)	-0.0150 to 0.0060
365 days	0.564 (0.339)	301 (57.1%)	0.576 (0.346)	286 (54.3%)	-0.016 (0.028)	-0.067 to 0.044	0.365 (0.200)	224 (46.3%)	0.365 (0.213)	228 (43.3%)	-0.0003 (0.0191)	-0.0378 to 0.0372
Total year							0.558 (0.265)	179 (34.0%)	0.535 (0.300)	182 (31.8%)	0.023 (0.030)	-0.036 to 0.081

SD, standard deviation; N, number; SE, standard error. ‡Difference between arms was calculated using t-tests

**Figure 1.**
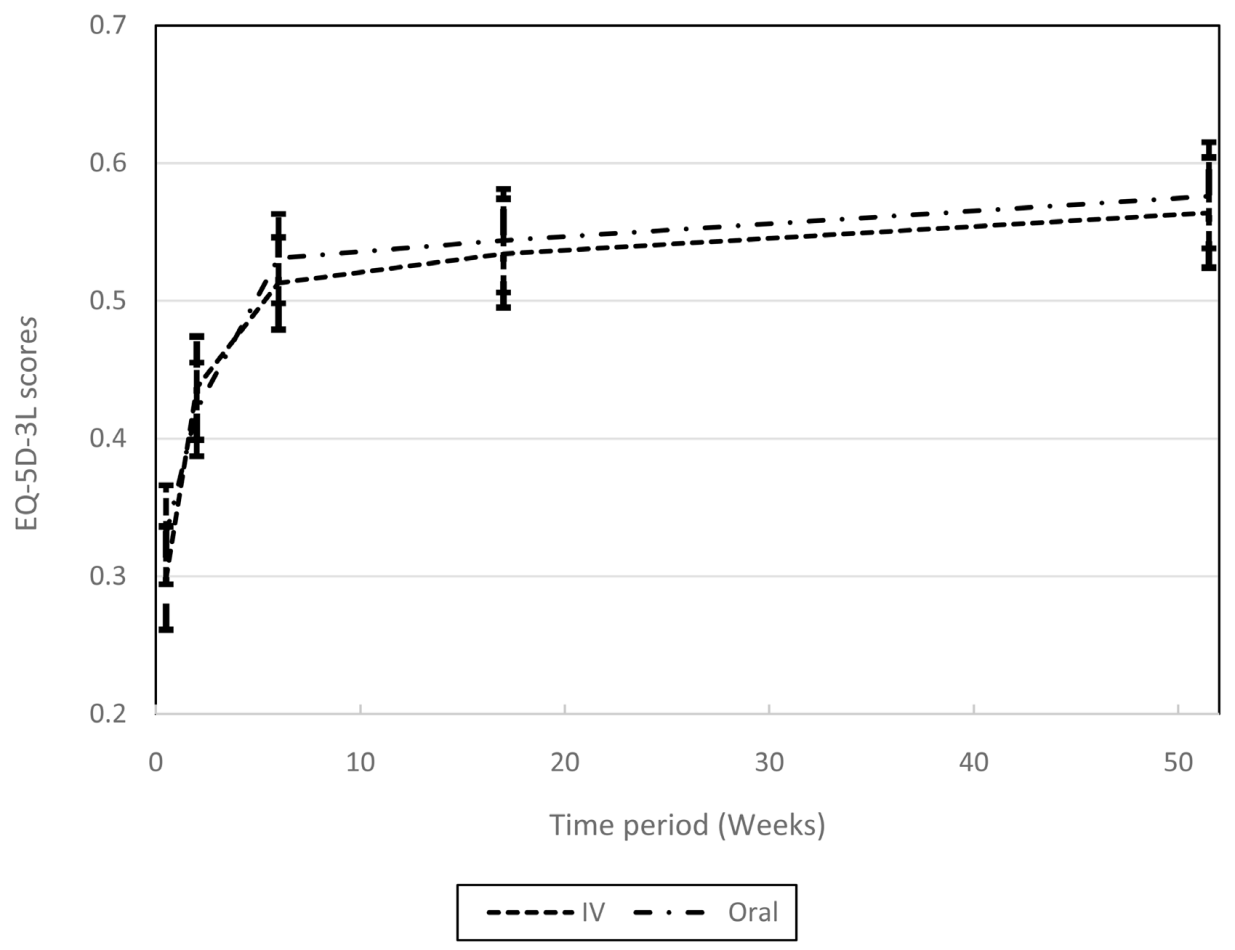
Complete case mean EQ-5D-3L utilities at baseline and follow-ups, with 95% confidence intervals.

The mean EQ-5D-3L utilities, along with 95% confidence intervals are presented in Figure 1.

Complete case QALYs results mirror those of the utilities with no statistically significant differences between arms at any follow-up point; intravenous 0.558 (SD 0.265) compared to oral 0.535 (SD 0.300). Results consider a zero-utility score for participants who died during the trial.

Multiple imputation results are provided in Table 6. These reflect the complete case results; there is no statistically significant difference in QALYs; however, the results now favour the oral arm.

**Table 6.** Multiple imputation results – total mean quality-adjusted life years (QALYs).

Intravenous mean QALYs (SE)	Oral mean QALYs (SE)	Difference (95% confidence interval)
0.537 (0.013)	0.545 (0.015)	-0.007 (-0.045 to 0.031)

SE, standard error

### Cost-effectiveness analysis

In the incremental analysis ( Table 7) base-case mean costs were observed to be lower in the oral arm and mean QALYs were higher in the oral arm, suggesting that the strategy of treating bone and joint infections with oral antibiotics is a dominant strategy (cheaper and with higher QALYs). The results of the sensitivity analyses indicate that the base-case conclusions were robust. Results for complete case, using mean imputation and altering the costs of OPAT were all consistent with the results from the base-case analysis; the total mean cost difference for all scenarios were within the range of £2,617 to £2,887. All of these results showed a statistically significant difference between arms. The results of multiple imputation and complete case QALYs show no statistically significant differences between arms. Uncertainty surrounding this result is explored further in the next section.

**Table 7.** Incremental cost-effectiveness results – base case and sensitivity analysis.

Analysis	Intravenous Mean costs (SE)	Oral Mean costs (SE)	Difference (95% confidence interval) ‡	Intravenous Mean QALYs (SE)	Oral Mean QALYs (SE)	Difference (95% confidence interval) ‡	Incremental cost per QALY
Base case (Multiple imputation)	£13,274 (£446)	£10,534 (£453)	£2,740 (£1,488 to £3,992)	0.537 (0.013)	0.545 (0.015)	-0.007 (-0.045 to 0.031)	Oral antibiotics dominant
Complete case	£13,275 (£10,113)	£10,549 (10,371)	£2,727 (£1,473 to £3,980)	0.558 (0.265)	0.535 (0.300)	0.023 (–0.036 to 0.081)	Oral antibiotics dominant
Mean imputation costs	£13,141 (£10,036)	£10,406 (£10,269)	£2,735 (£1,508 to £3,963)	0.537 (0.013)	0.545 (0.015)	-0.007 (-0.045 to 0.031)	Oral antibiotics dominant
District Nurse costs for all missing OPAT types	£13,274 (£448)	£10,657 (£463)	£2,617 (£1,354 to £3,880)	0.537 (0.013)	0.545 (0.015)	-0.007 (-0.045 to 0.031)	Oral antibiotics dominant
Self-administration costs for all missing OPAT types	£13,230 (£442)	£10,343 (£448)	£2,887 (£1,656 to £4,118)	0.537 (0.013)	0.545 (0.015)	-0.007 (-0.045 to 0.031)	Oral antibiotics dominant

QALYs, quality-adjusted life years; SE, standard error; OPAT, outpatient parenteral antimicrobial therapy. ‡Difference between arms was calculated using a generalized linear model

### Uncertainty

The main uncertainty in the results relates to QALYs; the difference in QALYs between arms is not statistically significant.

**Figure 2.**
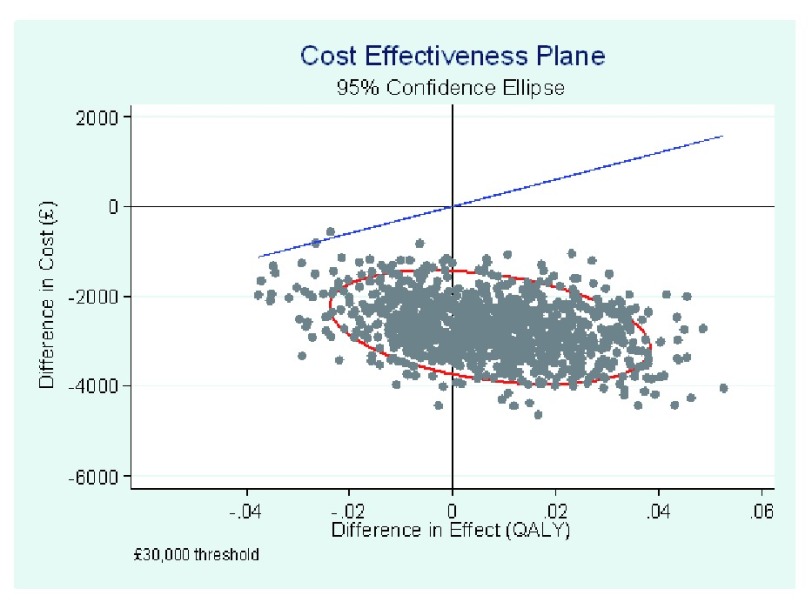
Cost-effectiveness plane.

The cost-effectiveness plane presented in Figure 2, shows 1,000 bootstrap samples of the ICER, along with a point estimate illustrating the mean differences in costs and QALYs between treatment arms. The graph also includes a 95% confidence ellipse from the bootstrap samples, and a line illustrating the £30,000 threshold currently used by NICE to assess cost-effectiveness ^9^. All bootstrap samples had a lower cost in the oral arm compared to the intravenous arm, and the majority of cost-effectiveness pairs fall into the south-east quadrant, where higher QALYs and lower costs can be observed for the oral arm as compared with the intravenous arm, making an oral intervention dominant for these samples.

## Discussion

### Statement of principal findings

The difference in costs between arms was £2,740 in the base case results; the use of oral antibiotics in the early treatment of bone or joint infection is significantly cheaper compared to the use of intravenous antibiotics. The results of the EQ-5D-3L questionnaires reflected the trial primary outcome of definitive failures; there was no statistically significant difference in QALYs between arms. This is reinforced by a post-hoc regression of QALYs on ‘definite failure’, which confirmed that the EQ-5D-3L measure is sensitive to the endpoint, but the endpoint did not differ between arms. With oral antibiotics being clinically non-inferior to intravenous, no statistically significant difference in QALYs plus the costs in the oral arm being significantly less than in the intravenous arm during the trial, the results of the trial suggest that treating patients with bone and joint infections with oral antibiotics is a dominant strategy.

There was no statistically significant difference in antibiotic duration in the post-intervention period suggesting that participants in the oral arm were not prescribed more antibiotics once finished on the intervention antibiotic. This is reflected by the difference between arms in the number of antibiotic prescriptions during the post intervention period not being statistically significant. As expected, the mean number of days that intravenous therapy was received during the intervention period was significantly higher in the intravenous arm; 28.22 days (95% confidence interval 24.66 to 31.77). Interestingly there was a significant difference in the post-intervention period also; 6.40 days (95% confidence interval 2.99 to 9.81). We found no significant difference in mean inpatient stay duration; however, there was a significant difference for median inpatient stay duration; 14 days (interquartile range 11 to 21) in the intravenous arm and 11 days (interquartile range 8 to 20) in the oral arm (p<0.001) ^8^.

Exploring uncertainty in the results using non-parametric bootstrapping, and for the bootstrap sample taken, there is a 100% probability that the oral strategy is cost saving. There is a 67% probability that the oral strategy results in higher QALY values than the intravenous strategy. This confirms prior evidence of clinical non-inferiority. Results from sensitivity analyses were consistent with the base case results.

A post-hoc analysis estimating mean costs for intravenous and oral antibiotics for a 42-day course out with the intention to treat population was conducted. The mean cost of a 6-week course of antibiotics (drug only) was £997 (SD £873) for intra- venous antibiotics and £188 (SD £648) for oral antibiotics, highlighting the higher costs for intravenous drugs.

### Strengths and limitations of the research

This is the first economic evaluation of oral versus intravenous antibiotics for treating bone and joint infections. The trial was a large inclusive, pragmatic trial with most participants following their allocated treatment and retention was high ^8^.

Some of the limitations arose from the high level of missing data for the EQ-5D-3L questionnaire (from 26.4% at baseline to 45.7% at 365-days). No costs of surgery for treatment of bone and joint infections were included in this study; this was a pre-randomisation procedure. Cost for insertion and removal of the intravenous line were obtained from clinical staff who had previously calculated the costs of insertion and removal, however we did not receive a detailed breakdown of the materials used, only time needed. However, given cost estimates were obtained from a reliable source, we believe that this will not impact our results. The data are likely to be skewed and the complete case results in Table 3, Table 4 and Table 5 should be viewed in this light. However, due to the large sample size the effect of the skewness will be moderate and generalised linear models were used in the main analysis.

### Strengths and weaknesses in relation to other studies, discussing important differences in results

Despite the high economic burden of bone and joint infections, economic studies in this area are rare ^22^ and there is a need for more economic evaluations of joint infections ^23^. No previous studies have explored the cost-effectiveness of oral antibiotics to treat bone and joint infections compared to intravenous antibiotics. A cost-effectiveness study, comparing exchange arthroplasty with debridement and prosthetic retention for infected total hip arthroplasty in the elderly, found debridement and retention improved quality-adjusted life expectancy and increased costs in 65- and 80-year-old men and women over a lifetime ^24^. The incremental cost-effectiveness ratio ranged from $500 for frail 80 year old men to $21,800 in 65 year old women. In an economic evaluation by Kapadia *et al*., the authors explored the use of chlorhexidine cloths prior to total knee arthroplasty and found that assuming 1,000 total knee arthroplasty patients a net saving of $2.1 million would occur ^25^. The study assumed an estimated cost of $130,000 per revision due to infection, with 22 patients in a cohort of 1,000 without use of the cloth becoming infected, and 6 infections in the cohort using the cloth. Two studies estimated revision costs for infected prostheses; for infected hips, estimated costs are £22,000 ^4^ and for infected knees, £30,000 ^5^. These costs included the revision surgery and subsequent inpatient stay. A 2013 review summarised the economic literature in the treatment of periprosthetic infections, looking at prevention, treatment and surgical options for periprosthetic infections ^26^. Unlike OVIVA, the treatment costs included the cost of revision and a 1993 study estimated an average cost of $50,000 to $60,000 per patient with an infected total hip arthroplasty ^22^.

### Meaning of the study

Annually in the UK, it is conservatively estimated that there are 6,350 post-operative bone and joint infections; if all of these were treated with oral antibiotics during the first six weeks of therapy there is a potential for savings to the NHS of around £17 million annually. The important benefits to patients receiving oral antibiotics compared to receiving intravenous antibiotics include a shorter median inpatient stay as well as decreased indwelling intravenous catheter days with associated reduced inconvenience, discomfort and complications ^27^. Ultimately, the savings made by the use of oral antibiotics in half of the trial participants have already exceeded the running costs of the clinical trial.

### Unanswered questions and future research

Further savings in the management of bone and joint infection might be possible by defining the optimal duration of therapy. At present, there are few trial data to guide duration and, in the opinion of the authors, there may be considerable redundancy in current standard treatment protocols. The benefits of limiting systemic antimicrobial exposure may well include a reduction in selection for antibiotic resistance and a consequent cost saving in managing treatment failures or transmission events.

What is already known on this topic:

- The ‘gold standard’ treatment for bone and joint infections is surgery followed by a course of intravenous antibiotics
- There is a growing body of literature showing that oral antibiotics are as effective as intravenous in treating this cohort
- Oral antibiotics are less costly than intravenous antibiotics

What this study adds:

- Oral antibiotics are non-inferior compared to intravenous antibiotics in treating bone and joint antibiotics with regards to definitive treatment failure
- Treating a bone or joint infection with an initial 6 weeks course of oral antibiotics saves an estimated £2,700 over one year, per person, compared to early treatment with intravenous antibiotics

## Data availability

### Underlying data

The ethical permissions governing this trial limit data sharing to approved studies of antibiotic treatment. Requests for participant level data should be directed to the chief investigator; Dr Matthew Scarborough (email address: Matthew.Scarborough@ouh.nhs.uk). Requests from interested parties will be granted access to the data when there is appropriate approval from their home institution for their analysis and where the purpose of the proposed analysis relates to antibiotic treatment, consistent with our ethical approval for sharing data

ClinicalTrials.gov number – NCT00974493 - ISRCTN91566927

### Reporting guidelines

Figshare: CHEERS checklist for “Cost-effectiveness of oral versus intravenous antibiotics (OVIVA) in patients with bone and joint infection: evidence from a non-inferiority trial”. https://doi.org/10.6084/m9.figshare.8197682.v1 ^28^.

The completed CHEERS checklist is available under the terms of the Creative Commons Attribution 4.0 International license (CC-BY 4.0).

## Author statement

NM, CG and AB conceived the presented idea. AB was co-investigator on the OVIVA project. NM carried out the analysis with input from CG and AB. NM and CG drafted the manuscript and AB, IR, HKL, PB, MM, BA, JF and MS contributed to the final version. MM conceived OVIVA, designed the protocol, obtained funding and recruited patients. PB conceived the OVIVA project, obtained funding, designed the protocol, recruited patients, gathered data and had general oversight of the OVIVA trial. MS was principle investigator of the OVIVA trial.

## References

[1] National Joint registry: National Joint Registry 15th Annual Report 2018 – HQIP.2018 Reference Source

[2] Public Health England: Surgical site infections (SSI) surveillance: NHS hospitals in England.2019 Reference Source

[3] Klouche S Sariali E Mamoudy P: Total hip arthroplasty revision due to infection: a cost analysis approach. *Orthop Traumatol Surg Res.* 2010;96(2):124–32. 10.1016/j.rcot.2010.02.005 20417910

[4] Vanhegan IS Malik AK Jayakumar P: A financial analysis of revision hip arthroplasty: the economic burden in relation to the national tariff. *J Bone Joint Surg Br.* 2012;94B(5):619–23. 10.1302/0301-620X.94B5.27073 22529080

[5] Kallala RF Vanhegan IS Ibrahim MS: Financial analysis of revision knee surgery based on NHS tariffs and hospital costs: does it pay to provide a revision service? *Bone Joint J.* 2015;97-B(2):197–201. 10.1302/0301-620X.97B2.33707 25628282

[6] Conterno LO Turchi MD: Antibiotics for treating chronic osteomyelitis in adults. *Cochrane Database Syst Rev.* 2013; (9): CD004439. 10.1002/14651858.CD004439.pub3 24014191 PMC11322802

[7] Li HK Scarborough M Zambellas R: Oral versus intravenous antibiotic treatment for bone and joint infections (OVIVA): study protocol for a randomised controlled trial. *Trials.* 2015;16:583. 10.1186/s13063-015-1098-y 26690812 PMC4687165

[8] Li HK Rombach I Zambellas R: Oral versus Intravenous Antibiotics for Bone and Joint Infection. *N Engl J Med.* 2019;380(5):425–36. 10.1056/NEJMoa1710926 30699315 PMC6522347

[9] National Institute for Health and Care Excellence: Guide to the Methods of Technology Appraisal: 2013. Reference Source 27905712

[10] Husereau D Drummond M Petrou S: Consolidated Health Economic Evaluation Reporting Standards (CHEERS) statement. *Eur J Health Econ.* 2013;14(3):367–72. 10.1007/s10198-013-0471-6 23526140

[11] Faria R Gomes M Epstein D: A guide to handling missing data in cost-effectiveness analysis conducted within randomised controlled trials. *Pharmacoeconomics.* 2014;32(12):1157–70. 10.1007/s40273-014-0193-3 25069632 PMC4244574

[12] British National Formulary Publications: BNF Publications.2016 Reference Source

[13] Department of Health and Social Care: NHS reference costs 2014 to 2015. Publications - GOV.UK.2016 Reference Source

[14] Wu O Boyd K Paul J: Hickman catheter and implantable port devices for the delivery of chemotherapy: a phase II randomised controlled trial and economic evaluation. *Br J Cancer.* 2016;114(9):979–85. 10.1038/bjc.2016.76 27092784 PMC4984916

[15] Curtis L: PSSRU | Unit Costs of Health and Social Care 2015.2016 Reference Source

[16] EuroQol Group: EuroQol--a new facility for the measurement of health-related quality of life. *Health Policy.* 1990;16(3):199–208. 10.1016/0168-8510(90)90421-9 10109801

[17] Dolan P: Modeling valuations for EuroQol health states. *Med Care.* 1997;35(11):1095–108. 10.1097/00005650-199711000-00002 9366889

[18] Billingham LJ Abrams KR Jones DR: Methods for the analysis of quality-of-life and survival data in health technology assessment. *Health Technol Assess.* 1999;3(10):1–152. 10.3310/hta3100 10627631

[19] Manca A Hawkins N Sculpher MJ: Estimating mean QALYs in trial-based cost-effectiveness analysis: the importance of controlling for baseline utility. *Health Econ.* 2005;14(5):487–96. 10.1002/hec.944 15497198

[20] Little RJA Rubin DB: The Analysis of Social Science Data with Missing Values. *Sociol Methods Res.* 1989;18(2–3):292–326. 10.1177/0049124189018002004

[21] Ramsey SD Willke RJ Glick H: Cost-effectiveness analysis alongside clinical trials II-An ISPOR Good Research Practices Task Force report. *Value Health.* 2015;18(2):161–72. 10.1016/j.jval.2015.02.001 25773551

[22] Fernandez-Fairen M Torres A Menzie A: Economical analysis on prophylaxis, diagnosis, and treatment of periprosthetic infections. *Open Orthop J.* 2013;7:227–42. 10.2174/1874325001307010227 24082966 PMC3785055

[23] Borgquist L W-Dahl A Dale H: Prosthetic joint infections: a need for health economy studies. *Acta Orthop.* 2014;85(3):218–20. 10.3109/17453674.2014.913227 24758324 PMC4062785

[24] Fisman DN Reilly DT Karchmer AW: Clinical effectiveness and cost-effectiveness of 2 management strategies for infected total hip arthroplasty in the elderly. *Clin Infect Dis.* 2001;32(3):419–30. 10.1086/318502 11170950

[25] Kapadia BH Johnson AJ Issa K: Economic evaluation of chlorhexidine cloths on healthcare costs due to surgical site infections following total knee arthroplasty. *J Arthroplasty.* 2013;28(7):1061–5. 10.1016/j.arth.2013.02.026 23540539

[26] Hernández-Vaquero D Fernández-Fairen M Torres A: Treatment of periprosthetic infections: an economic analysis. *Scientific World Journal.* 2013;2013:821650. 10.1155/2013/821650 23781163 PMC3679762

[27] Spellberg B Lipsky BA: Systemic antibiotic therapy for chronic osteomyelitis in adults. *Clin Infect Dis.* 2012;54(3):393–407. 10.1093/cid/cir842 22157324 PMC3491855

[28] McMeekin N: OVIVA CHEERS-Checklist.pdf. *figshare.*Figure.2019 10.6084/m9.figshare.8197682.v1

