# Supplementary material for: Cost-effectiveness of oral versus intravenous antibiotics (OVIVA) in patients with bone and joint infection: evidence from a non-inferiority trial
Source: Wellcome Open Res. 2020 Jan 8;4:108. Originally published 2019 Jul 17. [Version 4] doi: 10.12688/wellcomeopenres.15314.4 (PMC6944252; doi:10.12688/wellcomeopenres.15314.4)
Supplement: Peer review history

# Peer review: Reviewer response for version 3

**DOI:** 10.21956/wellcomeopenres.17138.r37435

**Published:** 2020-01-03

**Authors:** Simon Dixon

**Affiliations:** 1 Health Economics and Decision Science, School of Health and Related Research, University of Sheffield, Sheffield, UK

Thank you for the changes.

 Please note that the text relating to the cost-effectiveness plane needs amending as it still refers to confidence intervals. Also, the threshold is now missing from the Figure; it would be preferred if this were included.

I confirm that I have read this submission and believe that I have an appropriate level of expertise to confirm that it is of an acceptable scientific standard.

# Response

**Published:** 2020-01-07

**Authors:** Nicola McMeekin

**Affiliations:** University of Glasgow, UK

Thank you for your helpful comments and suggestions during the reviewing process, we appreciate the time you have taken to read and review this manuscript. In the latest version of the manuscript we have amended Figure 2 by including the £30,000 threshold line and updated the text so the 95% confidence intervals are now described as an ellipse.

# Peer review: Reviewer response for version 2

**DOI:** 10.21956/wellcomeopenres.17023.r36921

**Published:** 2019-11-26

**Authors:** Diarmuid Coughlan

**Affiliations:** 1 Health Economics and Evidence Synthesis Group, Institute of Health and Society, Newcastle University, Newcastle upon Tyne, UK

I am happy with the updates. I note that in Table 5 - you have N(%) and then you are inconsistent with the use of % in the table. Please correct formatting.

I confirm that I have read this submission and believe that I have an appropriate level of expertise to confirm that it is of an acceptable scientific standard.

# Response

**Published:** 2019-12-10

**Authors:** Nicola McMeekin

**Affiliations:** University of Glasgow, UK

Thank you for spotting the formatting inconsistency in Table 5, this has been corrected.

# Peer review: Reviewer response for version 2

**DOI:** 10.21956/wellcomeopenres.17023.r36922

**Published:** 2019-11-21

**Authors:** Simon Dixon

**Affiliations:** 1 Health Economics and Decision Science, School of Health and Related Research, University of Sheffield, Sheffield, UK

Thank you for making amendments in response to my comments. There are a few points that require a little more work. 

 In relation to point 5, your comments suggest that you do not know the full details of how the unit costs for insertion and removal were generated. This needs to be recognised as a limitation of the research; whether you consider this uncertainty to be important should also be stated and explained/justified.

 In relation to point 6, you have clarified that the bootstrapping did not account for covariate adjustment (and presumably the imputation of missing data). This needs to be clarified by saying that the bootstrapping is based on the unadjusted, non-imputed data. This is important as the adjustment and imputation can have a marked impact on the results, leading to a discrepancy between the bootstrapped and regression-based results.

 In relation to point 7, you have stated the type of test undertaken, but you have not justified this. The cost data are likely to be heavily skew (e.g. Table 4), the EQ-5D data are likely to be bimodal (e.g. Table 5) and the prescription data are likely to be count data (e.g. Table 3). Consequently, the appropriateness of the t-test is open debate.

 In relation to point 14, you very politely point out my incorrect interpretation of the 95% intervals on the ICER; thank you for that. What confused me is that it is very unusual for confidence intervals to be plotted on the cost-effectiveness plane. The use of confidence ellipses is the preferred method for summarising uncertainty in relation to a joint density function; this approach should be used here.

I confirm that I have read this submission and believe that I have an appropriate level of expertise to confirm that it is of an acceptable scientific standard, however I have significant reservations, as outlined above.

# Response

**Published:** 2019-12-10

**Authors:** Nicola McMeekin

**Affiliations:** University of Glasgow, UK

Many thanks for your useful comments, we feel that they have improved our paper.  In response to your comments we have made the following amendments to our manuscript:

We have added two points to the Limitation section:  units costs for insertion and removal, and the use of t-tests for comparing complete case results.

We have made it clear in the Methods section that the bootstrapping iterations are based on unadjusted, non-imputed data.

And finally we have revised the cost-effectiveness plane to include a confidence eclipse.

# Peer review: Reviewer response for version 1

**DOI:** 10.21956/wellcomeopenres.16715.r36165

**Published:** 2019-08-23

**Authors:** Diarmuid Coughlan

**Affiliations:** 1 Health Economics and Evidence Synthesis Group, Institute of Health and Society, Newcastle University, Newcastle upon Tyne, UK

General Comments:

 This is an important paper on the cost-effectiveness of oral versus intravenous antibiotics in patients with bone and joint infection. This is a very important clinical question as proven with the results of the trial published in the NEJM. It is also a very broad clinical question with nuance regarding types of infections, health status of patients and antibiotics used in the trial. 

 Some minor edits:

1. Table 1: be consistent with decimal point use (£190 vs £34.00).
2. Table 2: Not sure you need the Total column.  
3. Table 3 & 4: Labelling could be better. Place N beside Intravenous N=515 (97.7%) Oral N=513 (97.3%).
  Also, label days: Intervention period (Day 1-42) and post-intervention period (Day 43 -360)
4. Discussion section, paragraph: strengths and weaknesses in relation to other studies, discussing important differences in results.
  Typo: No previous studies have explored the cost-effectiveness of oral antibiotics to treat bone and joint infections compared to **oral (Should be intravenous?)** antibiotics.
5. Meaning of study - I would like to see a formal budget impact analysis. However, I realise that this would be a lot of additional work.  
6. I was confused by this statement:
  The important non-financial benefits to patients receiving oral antibiotics i **nclude a shorter median inpatient stay as well as decreased indwelling intravenous catheter days** with its associated inconvenience, discomfort and r **educed complications.**
  a) Table 3 reports non no difference in mean inpatient stays during the intervention and post-intervention period. Why mention median?
  b) Indwelling IV catheter complications - was this is an issue in the clinical trial? If so, please elaborate.
7. Please add the original trial registration information in the data availability section:
  - ClinicalTrials.gov number
  - NCT00974493
  - ISRCTN91566927 
8. I agree with many of the comments made by the other reviewer (Simon Dixon). Don't think Table 5 is necessary. I would try to cut down on some of the details in the other tables and put into a supplemental appendix. (e.g. 'Total' section of Table 3). Table 4 could be more parsimonious using a 'Landscape' table with 3 rows for the cost category and columns for intervention and post-intervention period stratified by IV and oral. Table 9 needs a better label.
9. Statement on Covariate adjustment is a good point made by Simon.  

Overall, it's a good paper. I think 9 Tables and 2 Figures in the main manuscript is too much and distracting for a broad clinical/scientific audience.

I confirm that I have read this submission and believe that I have an appropriate level of expertise to confirm that it is of an acceptable scientific standard, however I have significant reservations, as outlined above.

# Response

**Published:** 2019-10-29

**Authors:** Nicola McMeekin

**Affiliations:** University of Glasgow, UK

Thank you for taking the time to review this manuscript.  We address your comments below:

 1) Thank you, we have changed this in the table.

 2) We are happy to delete the Total column if the reviewer deems this necessary.

 3) Thank you, we have changed this in both tables.

 4) Changed, thanks you!

 5) Whilst we agree that this might add value to our study, we feel that this would be beyond the scope of our study and would require substantial additional data collection and analysis and almost merit a stand-alone paper. We hope that the reviewer might agree with us on this point.

 6) Thank you, we have made this sentence clearer and added a reference.

 6a) Median was mentioned in the NEJM paper, so we have included it to be consistent with that.

 6b) No, this issue was not added as a result of the trial, but a discussion into the benefits of oral antibiotics, a reference has been added to clarify this.

 7) We have added this, thank you.

 8) Table 5 has been removed and tables 6 and 7 (EQ-5D and QALY results) have been combined in Table 5. If necessary we could put the unit cost table – Table 1 in an a supplementary appendix, if that is possible with Wellcome Open Research. We have changed the label for Table 9 (now Table 7).

 9) Thank you, we have included more detail in the manuscript - please see the reply to Simon's comment.

# Peer review: Reviewer response for version 1

**DOI:** 10.21956/wellcomeopenres.16715.r36050

**Published:** 2019-08-12

**Authors:** Simon Dixon

**Affiliations:** 1 Health Economics and Decision Science, School of Health and Related Research, University of Sheffield, Sheffield, UK

General comment

 The work is undertaken to a high standard, is clearly written and produces robust conclusions. The research is very important.

 Some relatively minor details are omitted and need to be added.  Some methods deviate from the ‘standard approach’, and as such, need to be justified more clearly.

 Specific comments

1. An undefined personal communication is used for an important estimate. Either something should be said about its source (e.g. opinion or audit) or a published estimate should be used [first paragraph, p3].
2. “Missing resource and quality of life data was imputed….”, should be “…were imputed” [middle paragraph, second column, p3].
3. More should be said in the Introduction or Methods about how IV and oral antibiotics are administered and the role of the OPAT team. This is important for readers to assess the generalisability of findings. It is also unclear how you can have an “inpatient” OPAT type [p4]; I thought OPAT was outpatient.
4. “…an appropriate method to replace missing data utilised”. On what basis was appropriateness assessed? Just giving a reference is insufficient. Also, I would have thought that Reference 11 would have been used for this assessment, but it is not referenced at this point. [p4]
5. In Table 1, simply saying that “insertion” and “Removal” costs were based on expert opinion is insufficient. Presumably, the experts didn’t come up with the cost; they provided timings, staff grades and consumables, which were then costed up. Those resource estimates should be provided, possibly as notes to the table. [p4]
6. It is stated that QALYs were adjusted for baseline utility, but not how this was done; providing a reference is insufficient. If this was done statistically, which is implied by the reference, how did you then generate the bootstrapped estimates? [p4]
7. The statistical tests used to compare groups in all the tables are not described. These need to be justified.
8. The inpatient duration for oral therapy is almost identical to that for IV. An explanation of this needs to be given. A lay reader may equate oral therapy to home treatment and IV therapy to inpatient treatment, therefore inpatient duration may be expected to be similar to number of days of IV therapy. [p6]
9. The sentence starting “Table 4 presents….” should be the first sentence of the next paragraph.
10. An exploratory analysis appears in the middle of the results, would this be better framed as a sensitivity analysis? The way it is presented suggest that it is unplanned/post-hoc.
11. Is Table 5 necessary? The results are given in the text anyway (except for the estimates of variation, which could be added). [p8]
12. The post-hoc regression of QALYs on failure is not a result; it should be part of the discussion about the perceived weakness of the EQ-5D for this condition/study. [p9]
13. The description of the bootstrapped replications in cost-effectiveness plane on [p9] and [p11] don’t seem to match the figure, for example 82.8% fall into the south east quadrant. Do you mean, fall to right of the cost-effectiveness threshold line? You also say that 17.2% of the sample result in higher QALYs, really?
14. The threshold line in the SE quadrant is not necessary. [p10]
15. The statement starting “There is a 100% probability that...” includes an ‘and statement’ which makes interpretation confusing/difficult. This needs to be re-written. You may also want to consider qualifying the statement by saying that it relates to this particular set of bootstrapped samples, another set may produce a lower probability. [p11]
16. Something needs to be said about covariate adjustment within the economics analysis. Whilst an adjustment has been made for baseline utility (but not described sufficiently), it is common in economic evaluation for other covariates to be included in a regression based analysis, sometimes in the form of a related regression on QALYs. Whilst I don’t consider this to be a big issue in this instance, the authors should describe this alternative approach and explain the appropriateness their approach, this is methodological uncertainty.
17. Something needs to be said about the truncation of data. Alternative methods would be to take account of this within a regression analysis of costs and QALYs using appropriate specifications, or to extrapolate the costs/QALYs. Again, whilst I don’t consider this to be a big issue in this instance, the authors should describe this alternative approach and explain the appropriateness of their approach, this is methodological uncertainty.

I confirm that I have read this submission and believe that I have an appropriate level of expertise to confirm that it is of an acceptable scientific standard, however I have significant reservations, as outlined above.

# Response

**Published:** 2019-10-29

**Authors:** Nicola McMeekin

**Affiliations:** University of Glasgow, UK

Thank you for taking the time to comment on our manuscript.  We address each of your points in our response below:

 1) Thank you - we have added 'opinion' to clarify this point.

 2) Thank you, we have changed this.

 3) We have expanded on this on the information in paragraph 3 on the administration of IV and oral antibiotics.

 The different types of OPAT collected on the CRF were:

- Self-administering                                                                                                  
- District nurse administering                                                                                
- Attending clinic for administration                                                                   
- In intermediate or long-term care facility administering antibiotics    
- Completed IVs as inpatient                                                                                 
- Not on IVs, PO on discharge only

That is why there is an ‘inpatient’ OPAT type.

 4) Thank you, we have moved reference 11 to the sentence before in the manuscript as suggested and rewritten this sentence.

 5) Thank you – we have added more detail to the table. The expert had already computed the cost for other purposes so we didn’t get a full breakdown to include.

 6) Thank you, this has been clarified in the manuscript, there was no adjustment for baseline utilities in the bootstrapping sample.

 7) T-tests were used to compare differences for continuous measures between groups in complete case analysis. For MI results we used a glm model. This is now being mentioned in the manuscript.  

 8) The median difference is different from the mean, the median difference is 14 v 11 days, this was a statistically significant difference, and mentioned in the Discussion section. Also, the trial protocol allowed for participants to have an initial maximum of 7 days treatment before being randomised so many were on IV antibiotics initially. The standard deviation for the mean in the oral arm was larger than that for the IV arm showing wider variability in this arm compared to the IV arm.  Finally, there is likely to be a number of reasons that a participant would require an inpatient stay other than IV antibiotics.

 9) Thank you, we have changed this.

 10) Thank you, this analysis was carried out to confirm that a course of 42 days intravenous antibiotics is more expensive than 42 days oral antibiotics, and has been moved to the Discussion section as a post-hoc analysis.

 11) This has been deleted, thank you.

 12) Thank you, this has been deleted from results and moved to the Discussion section.

 13) Thank you – we have amended the paper.

 14) Thank you, this is not a threshold line, it is a line that depicts the upper 95% confidence interval.

 15) Thanks, this sentence has been amended.

 16) We have amended the ‘Data analysis’ section to include the covariates used in the adjustment.

 17) Censored but not dead participants had costs and QALYs extrapolated in the MI adjustment, we have included a sentence to explain this in the Methods section.
